# A Rare Clinical Course of Seronegative Pulmonary-Renal Syndrome

**DOI:** 10.1155/2016/4893496

**Published:** 2016-10-27

**Authors:** M. Fröhlich-Gildhoff, W. J. Jabs, C. Berhold, M. K. Kuhlmann, U. Ketterer, S. Kische, H. Ince

**Affiliations:** ^1^Department of Cardiology and Intensive Care Medicine, Vivantes Klinikum im Friedrichshain, Landsberger Allee 49, 10249 Berlin, Germany; ^2^Department of Nephrology, Vivantes Klinikum im Friedrichshain, Landsberger Allee 49, 10249 Berlin, Germany

## Abstract

*Purpose*. Pulmonary-renal syndrome (PRS) is characterized by diffuse alveolar hemorrhage and rapidly progressive glomerulonephritis mainly due to autoimmune etiologies. Seronegative PRS is a challenging entity to the clinician, since early diagnosis may be missed leading to delayed appropriate treatment.* Materials and Methods*. We present the clinical course of a 77-year-old patient who was admitted under the suspected diagnosis of pneumogenic sepsis and septic renal failure with fever, dyspnea, and elevated CRP levels. The diagnosis of pulmonary-renal syndrome was initially missed because of the absence of autoantibodies in all serological findings.* Results.* Despite delayed initiation of immunosuppressive therapy and a prolonged period of dialysis and extracorporeal membrane oxygenation the patient recovered well and was released to a rehabilitation center with nearly normalized creatinine levels. The diagnosis of PRS was established by renal biopsy.* Conclusion*. This case illustrates the important differential diagnosis of seronegative pulmonary-renal syndrome in patients with pulmonary and renal impairment.

## 1. Introduction

Pulmonary-renal syndrome (PRS) is a rare clinical condition defined by rapidly progressive glomerulonephritis (RPGN) and diffuse alveolar hemorrhage (DAH). Its etiology is mainly autoimmune and can be categorized by serological findings (ANCA; anti-GBM antibodies) and renal or pulmonary biopsy. In over 90% of the cases autoantibodies (ANCA, anti-GBM) can be detected, making unclassified PRS a diagnostically challenging entity to the clinician.

We hereby present a case in which the diagnosis of seronegative PRS was established by a pauci-immune RPGN in renal biopsy, in a patient that had undergone immunosuppressive treatment eight months before admission to our hospital due to PRS, positive for both ANCA- and anti-GBM antibodies.

## 2. Case Report

We report the case of a 77-year-old German woman, who presented to our emergency department due to dyspnea, cough, hemoptysis, and fever.

Initial vital signs were as follows: RR 165/60, heart rate 89 bpm, SpO_2_ 96%, respiratory rate 28/min, and temperature 38.8°C.

Her past medical history included Goodpasture's syndrome, chronic obstructive pulmonary disease, smoking, high blood pressure, diabetes mellitus type II, and hyperlipidemia.

Eight months before admission the patient had suffered a dialysis-dependent acute kidney failure and an acute respiratory distress syndrome with long-term ventilation, caused by diffuse alveolar hemorrhage. Diagnosis of Goodpasture's syndrome was established by positive serological tests for anti-GBM antibodies and responded well to immunosuppressive therapy with corticoids and cyclophosphamide. Renal biopsy had not been performed, although ANCA also had been tested positive and therefore polyangiitis would have been a likely differential diagnosis. Due to persistent leucopenia and reoccurring infections cyclophosphamide therapy was stopped four months before admission to our hospital and had been replaced by 10 mg prednisolone per day.

On admission to our hospital laboratory results showed an elevation of C-reactive protein level (CRP 94 mg/L) and moderate renal impairment (creatinine 1,3 mg/dL, GFR 38 mL/min) without alterations in white or red blood cell count. Pulmonary radiography showed opacities in the left lower and middle lung zone and antibiotic treatment (Piperacillin/Tazobactam, Ciprofloxacin) was initiated under the suspected diagnosis of pneumonia under immunosuppression.

After progressive worsening of the patient's status with continuous dyspnea even under noninvasive ventilation (FiO_2_ 1.0), a further increase in CRP (264 mg/L) under escalated antibiotic treatment (vancomycin, Clarithromycin, and Voriconazole), and deteriorated renal function (creatinine 2.7 mg/dL), she was admitted to our intensive care unit on day 6 after hospitalization.

**Figure 1:**
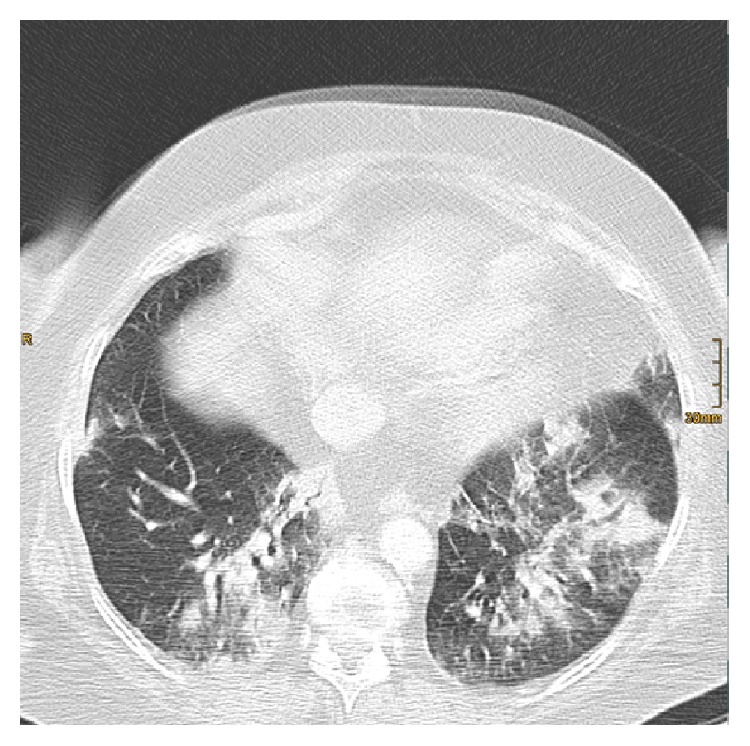
Illustrating diffuse bilateral infiltrates of the lung.

A chest-CT scan showed progressive, diffuse bilateral infiltrates, corresponding to a further decline of the respiratory situation and leading to intubation on day 12 (see Figure 1). Bronchoscopy, performed on day 13, showed signs of pulmonary hemorrhage with microbiological findings of* C. albicans* and no abnormal results in cytological testing.

Laboratory testing for Goodpasture's syndrome (anti-GBM ELISA, Alegria®, Orgentec) on days 5, 26, and 49 as well as vasculitis (MPO-ANCA, PR3-ANCA; ELISA Orgentec and ANAM; immunofluorescence) on days 13, 26, and 49 repeatedly showed negative results. The negative test results hereby differed from the serological testing in the patient's fulminant Goodpasture's syndrome which had occurred eight months earlier.

A further deterioration in pulmonary function with failing oxygenation under highly invasive ventilation protocols resulted in the application of a system of extracorporeal membrane oxygenation (ECMO, CARDIOHELP®, Maquet) on day 16; tracheotomy had been performed on day 26.

Failing kidney function required the initiation of continuous venovenous hemodialysis (CVVHD) from day 25. Urinary testing showed unselective proteinuria of 2300 mg/g creatinine (of which albumin 1462 mg/L) consistent with glomerular damage and mild hematuria (erythrocytes 97/*μ*L) as well as leukocyturia (50/*μ*L). Microscopic exploration showed hyaline cylinders.

In spite of high CRP levels (290 mg/L) we suspected a relapse of a pulmonary-renal syndrome with pulmonary hemorrhage rather than an infection because of low PCT levels and a drop of hemoglobin from 12.6 to 8.1 g/dL within 8 days due to alveolar bleeding. Moreover, the patient showed no clinical improvement under long-lasting broad antibiotic treatment.

Despite repeatedly negative results in laboratory testing for anti-GBM and ANCA a therapy with methylprednisolone (1 g/d) was started on day 26, continued for three days, and followed by a therapy with prednisolone 80 mg per day. It was accompanied by the start of plasma exchange for the duration of 6 days as well as the application of 750 mg of cyclophosphamide on day 31. Under this regime of immunosuppressive therapy the pulmonary and renal function rapidly improved leading to the termination of ECMO therapy and the alternation from CVVHD to intermittent hemodialysis on day 33, which had to be continued until day 50. CRP levels also dropped significantly from 290 to 36 mg/L.

The course of the patient was then complicated by a pneumogenic septic shock with the detection of MRSA in a bronchoalveolar lavage and blood cultures. The situation was stabilized under an escalation of the antibiotic regime to vancomycin and imipenem.

After septic thrombopenia and the impaired hemostatic situation had been improved, renal biopsy was performed on day 44 and revealed scarred intra- and extracapillary glomerulonephritis (crescents) of the pauci-immune type, coherent with rapidly progressive glomerulonephritis and polyangiitis. It also showed diffuse potentially reversible tubular damage and mild benign nephrosclerosis. Despite repetitive dilutions no linear deposition of IgG was observed on the basement membrane by immunofluorescence microscopy.

After the diagnosis of RPGN had histologically been established immunosuppressive therapy was continued with MabThera (Rituximab 1000 mg on days 65 and 79).

Under antibiotic and immunosuppressive therapy the patient's status continually improved. The mobilization of the patient, which initially was complicated by a proximal tetraparesis due to critical illness myopathy (MRI-scan on day 53 showed no signs of myelopathy), eventually showed progress and she could be released to a rehabilitation center on day 104 after admission with a maintenance dosage of prednisolone of 15 mg/d.

Pulmonary function had normalized entirely by the date of discharge. The kidneys showed only moderate impairment (creatinine 1.27 mg/dL; GFR 41 mL/min). The urinalysis showed persistent proteinuria of 888 mg/g creatinine with no further hematuria, probably due to scarred glomeruli.

## 3. Discussion

Pulmonary-renal syndrome (PRS) is rare and describes the clinical cooccurrence of renal impairment due to glomerulonephritis and a decrease in pulmonary function due to diffuse alveolar hemorrhage. The underlying causes of PRS are heterogeneous and contain a group of autoimmune disorders, which are mainly classified by the detection of antibodies, renal or lung biopsy, or cytological findings in bronchoalveolar lavage. They mainly include ANCA-associated small vessel vasculitis (such as Wegener's granulomatosis, microscopic polyangiitis, and Churg-Strauss vasculitis), Goodpasture's syndrome, systemic lupus erythematosus, Henoch-Schönlein purpura, or cryoglobulinemia. Other rare causes of PRS include drug-induced vasculitis (e.g., propylthiouracil) and subacute endocarditis.

As a particularly deleterious cause for PRS Goodpasture's syndrome is defined by the occurrence of antibodies against the alpha3-NC1-domain of collagen IV in the glomerular or alveolar basement membrane and typical linear deposition of IgG (rarely IgA) observed in part of the basement membrane by immunofluorescence microscopy.

Studies suggest that over 90% of patients with PRS present with one or more antibodies (ANCA, anti-GBM) in the serum, making PRS with no detection of antibodies as in the above-mentioned case very rare [1–3], albeit there have been reports of seronegative relapses in anti-GBM disease after immunosuppressive therapy [4].

In this case two differential diagnoses for the underlying clinical condition seemed possible: a seronegative relapse of anti-GBM disease—which initially had only been diagnosed by circulating antibodies but without renal biopsy—as well as a seronegative ANCA-vasculitis with RPGN and diffuse alveolar hemorrhage.

In the renal biopsy, scarred crescents without linear deposition of IgG along the basement membrane emphasize the possibility of a seronegative ANCA-vasculitis with RPGN. A significant improvement of renal function as in our patient seems to make the diagnosis of an ANCA-associated vasculitis more probable, as anti-GBM diseases like Goodpasture's syndrome usually lead to severe renal impairment and long-term dialysis [5, 6], particularly when treatment is delayed. On the other hand, there have been suggestions that a seronegative anti-GBM disease with predominant pulmonary involvement, as in our case, might be more common than generally suspected and case reports of seroconversion to seronegativity in anti-GBM diseases treated with immunosuppression exist [4]. Additionally, a correlation between cigarette smoking and pulmonary hemorrhage in patients with anti-GBM disease has been demonstrated by Donaghy and Rees [7].

Even though testing by commercially available ELISA-kits, as in our case, is generally considered to be a highly sensitive method for the detection of anti-GBM, the possibility of existing anti-GBM in very low concentrations remains. Furthermore, no tests other than ELISA (e.g., immunoblot, indirect immunofluorescence) have been performed. Importantly, an incidence of 2-3% of seronegative anti-GBM has been estimated by the British reference laboratory for anti-GBM antibodies [8].

The prognosis of PRS derives from simple center experience with low patient numbers. Gallagher et al. observed a 36% early mortality in their single-center study (*n* = 14) with a mean follow-up of four years [9]. Predictors of poor prognosis are crescents in >50% of glomeruli in renal biopsy, serum creatinine levels >5-6 mg/dL, oliguria, or the need for acute dialysis on admission. The early establishment of the correct diagnosis followed by prompt immunosuppressive therapy and plasma exchange are pivotal points in the treatment of PRS.

Diagnosis was missed early in our case due to the complete absence of antibodies, delaying renal biopsy which was performed after immunosuppressive therapy had been started on day 26. The observation of almost complete convalescence of renal and pulmonary function despite delayed disease-specific treatment makes this case very interesting.

Clinical data concerning the course of patients with seronegative PRS is scarce and in most cases the disease leads to end-stage renal failure [1, 10, 11]. As in PRS with a detection of specific antibodies or typical findings in renal or pulmonary biopsy, treatment with corticoids and cyclophosphamide is the basis of any therapy for patients with a suspected seronegative PRS. In some cases plasma exchange has been described to have beneficial effects on patients with seronegative and therefore uncategorizable PRS [12].

## 4. Conclusion

Seronegative pulmonary-renal syndrome is a rare but potentially life-threatening clinical condition. As the diagnostic process is complicated by the absence of antibodies, end-stage renal disease and even death have been reported as a common consequence of seronegative PRS. This case illustrates the rare instance of renal and pulmonary recovery after therapy with corticosteroids, cyclophosphamide, plasma exchange, and Rituximab, notwithstanding the temporary necessity for renal and pulmonary supportive therapy by dialysis and ECMO.
